# Effects of regulated learning scaffolding on regulation strategies and academic performance: A meta-analysis

**DOI:** 10.3389/fpsyg.2023.1110086

**Published:** 2023-03-22

**Authors:** Jingjing Shao, Yunshan Chen, Xiaoyang Wei, Xiaoran Li, Yanyan Li

**Affiliations:** ^1^Faculty of Education, Beijing Normal University, Beijing, China; ^2^Beijing Advanced Innovation Center for Language Resources, Beijing Language and Culture University, Beijing, China

**Keywords:** regulated learning scaffolding, SRL, SSRL, regulation strategies, academic performance, meta-analysis

## Abstract

Education research is increasingly focused on fostering self-regulated learning (SRL) and socially shared regulation of learning (SSRL) among students. However, previous meta-analyses have rarely focused on the specific types of regulated learning scaffolding. Therefore, this meta-analysis examines the effects of different types of regulated learning scaffolding on regulation strategies and academic performance. A total of 46 articles met the inclusion criteria and were included in the final analysis. The findings showed that overall, regulated learning scaffolding had a moderate effect (*g* = 0.587). In addition, moderation analyses were performed using a random effects model that focused on four types of scaffolding. The results showed that overall, composite tools had the greatest effect, while the most useful scaffolding for SRL and SSRL were group awareness tools (*g* = 0.61) and composite tools (*g* = 0.53), respectively. In terms of learning outcomes, composite tools had the greatest effect on regulation strategies, while intelligent pedagogical agents had the greatest effect on academic performance. We also performed a meta-regression analysis to identify the moderators that had the greatest influence on the effects of regulated learning scaffolding. The results showed that grade level, academic subject, and cooperation all had a significant impact. In conclusion, these findings provide evidence for validating the effectiveness of four regulated learning scaffolding and for discovering their function for SSRL, and presented some practical implications of our findings.

## 1. Introduction

Self-regulated learning (SRL) is a crucial element in the student learning process (Jansen et al., 2019) that is essential for the cultivation of students’ lifelong learning competence and employability (Bruijn-Smolders et al., 2016; Theobald, 2021). Students must regulate their own behavior and cognition effectively and in a timely manner if they are to achieve positive learning outcomes (Duffy and Azevedo, 2015). In addition, meaningful learning requires group members’ active interaction and the co-construction of shared goals and strategies (Zheng et al., 2017; Zabolotna et al., 2023). Therefore, it is necessary to focus on not only individual learning but also socially shared regulation of learning (SSRL) (Hadwin et al., 2011; Zheng et al., 2017). Rogat and Linnenbrink-Garcia (2011) identified the strong link between SRL and SSRL, and highlighted the contextualized nature of students’ experiences during shared activities. Previous studies have also shown that high levels of SSRL are associated with reduced social loafing and improved problem-solving (Panadero and Järvelä, 2015), and play a critical role in collaborative learning (Zheng et al., 2017).

However, students often lack the necessary regulated learning knowledge, and fail in their SRL and SSRL (Lin, 2018). For instance, they are unable to manage self-regulation processes and activities spontaneously (Bannert and Reimann, 2012), perform poorly in terms of time and study management (Theobald, 2021), and have difficulty in collectively regulating cognitions, emotions, metacognitions, and behaviors (Zheng et al., 2017). Hence, it is necessary to use scaffolding to support regulated learning (including SRL and SSRL), given that SRL and SSRL have a mutual influence on collaborative learning (Grau and Whitebread, 2012). Despite a wealth of empirical studies exploring the effects of various scaffolding on regulation strategies and academic performance (Troussas et al., 2021; Azevedo et al., 2022), such as scripts (Chen et al., 2014; Raes et al., 2016), intelligent pedagogical agents (Duffy and Azevedo, 2015), and group awareness tools (Lin, 2018), there is no consensus about the scaffolding’s effects. Therefore, it is necessary to examine the functions of various regulated learning scaffolding using meta-analysis. Moreover, previous meta-analysis has not yet focused on the type of regulated learning scaffolding, i.e., the macro level (the type of regulated learning scaffolding) rather than the micro level (such as functions, delivery forms, and so on). This research intended to focus on four regulated learning scaffolding and explored their respective effects at a macro level. Further, although there were many meta-analyses on SRL scaffolding, those on SSRL scaffolding remained scarce. Scholars have not previously verified the function of the regulated learning scaffolding on SSRL using meta-analysis. Thus, the first goal of this meta-analysis was to investigate the overall effectiveness of regulated learning scaffolding. The second goal was to explore the specific effects of scaffolding on the type of regulated learning (SRL/SSRL) and learning outcomes (regulation strategies and academic performance). The third goal was to identify the factors influencing the effectiveness of various scaffolding.

## 2. Literature review

### 2.1. Self-regulated learning and socially shared regulation of learning

SRL is defined as an “active, constructive process whereby students set goals for their learning and then attempt to monitor, regulate, and control their cognition, motivation, and behavior, guided and constrained by their goals and the contextual features of their environment” (Pintrich, 1999). Students who engage in SRL take control of their own learning process (Jansen et al., 2019), which can generally be divided into three phases: preparation, performance, and appraisal (Panadero et al., 2017). Learners analyze the task and set goals in the preparation phase, supervise and control the learning process in the performance phase, and reflect on the process to facilitate subsequent learning in the appraisal phase (Theobald, 2021).

Socially shared regulation of learning has attracted increasing attention with the enrichment of collaborative learning scenarios and tools. Collaborative learning provides opportunities for shared knowledge construction and productive interactions (Dillenbourg, 1999). Shared regulation occurs when groups of learners regulate their learning together, such as when they construct shared task perceptions or shared goals, and thus SSRL can be defined as a process in which a group of learners co-construct plans or align their monitoring perceptions to establish a shared evaluation of learning (Järvelä et al., 2013), regarding learning as the co-construction of knowledge.

Numerous researchers have found empirical evidence of SRL as a widespread social phenomenon (e.g., Volet et al., 2009; Järvelä et al., 2013), and previous review studies have consistently shown that SRL is related to higher levels of student achievement (Dignath et al., 2008; Sitzmann and Ely, 2011; Boer et al., 2014). Numerous studies of SSRL have focused on how groups regulate their collaborative work and how this affects their learning experience as a group (Järvelä et al., 2013; Panadero and Järvelä, 2015), and have found that the type of regulation that develops over time is related to the degree of collaborative success. Thus, SRL and SSRL have become important topics in current research.

### 2.2. The effects of scaffolding on the type of regulated learning and learning outcomes

The current meta-analysis focusses on regulated learning scaffolding. Scaffolding can be defined as the process of supporting learning efforts in an open learning environment (Zheng, 2016). They can be platforms, scripts or tools (Troussas et al., 2013; Zheng, 2016; Lin, 2018; Krouska et al., 2019). In this regard, “regulated learning scaffolding” refers to the process through which self-regulated learning and socially regulation of learning efforts are supported. In recent years, more and more researchers have focused on regulated learning scaffolding to facilitate students’ regulating strategies and academic performance (Janssen et al., 2007; Lin, 2018; Yilmaz-Na and Sönmez, 2023). Among these, four types of scaffolding can be classified based on their functions: scripts (Azevedo et al., 2004), group awareness tools (Lin et al., 2016; Lin, 2018), intelligent pedagogical agents (Duffy and Azevedo, 2015; Jones et al., 2018), and composite tools (Janssen et al., 2007; Zheng et al., 2017). Scripts are scaffolds that provide collaborators with task-related interactive instructions, which can be represented in different ways and tailored to specific learning objectives, and can implicitly or explicitly specify the collaboration roles and the sequences of activities (Kollar et al., 2006). Group awareness tools provide tacit guidance in understanding group members’ learning activities, participation status, and contributions by visually presenting member activities to other group members or teams in a computer-supported collaborative learning (CSCL) environment (Lin, 2018). An intelligent pedagogical agent is a virtual agent that is embedded in a computer-based learning environment and provides instruction through verbal and non-verbal forms of communication using images of animated or human-like figures (Lin et al., 2020). Composite tools are those that combine two or more types of scaffolding. Different types of regulated learning scaffolding have different delivery modes, and can be either direct or indirect, fixed or adaptive, hard or soft, and embedded or non-embedded (Devolder et al., 2012).

Numerous studies have found that regulated learning scaffolding can improve students’ academic writing (Teng, 2022), monior and understand their learning (Moos and Azevedo, 2008), and enhance regulation strategies (Lin, 2018). For instance, Azevedo et al. (2004) pointed out that adaptive scaffolding can regulate students’ learning by activating prior knowledge, monitoring their understanding using various strategies, and engaging in adaptive assistance. Duffy and Azevedo (2015) found that students’ use of self-regulatory strategies was significantly improved by the support of an intelligent pedagogical agent. However, other studies have suggested that regulated learning scaffolding does not always work as well as expected, and might have little influence on students’ learning (Malmberg et al., 2015; Raes et al., 2016). For example, Malmberg et al. (2015) showed that a low-performing team failed to identify the challenge using learning tools. Similarly, Raes et al. (2016) found that a collaborative script only had a marginal effect on socially shared regulation. Scholars have also found that regulated learning scaffolding has inconsistent effects on SRL and SSRL. For example, Lin (2018) found that the experimental group (group awareness) and the control group did not differ significantly in relation to unbalanced SSRL, but differed noticeably in relation to SRL. Similarly, Teng (2022) and Manlove et al. (2006) reported contrasting findings regarding the role of scripts, with Teng (2022) finding that scripts did not have a significant effect on SRL, while Manlove et al. (2006) identified a positive effect of scripts on SSRL. Therefore, there is a lack of consensus on the effects of regulated learning scaffolding, raising the question of what relationships exist among regulation strategies, academic performance, and regulated learning scaffolding. Thus, a meta-analysis is needed.

Some previous meta-analyses have examined the effects of regulated learning scaffolding on academic performance and regulation strategies and found small to medium effects. For example, Guo (2022) examined the effects of metacognitive prompts on students’ self-regulated learning (SRL) and learning outcomes. Results found that metacognitive prompts significantly enhanced SRL activities (*g* = 0.50) and learning outcomes (*g* = 0.40). Given the importance of specific regulation strategies on student academic performance varies (Theobald, 2021), however, Guo’s meta-analysis did not spotlight specific levels of regulation strategies. Theobald (2021) further focused on the relationship between regulated learning scaffolding and specific regulation strategies, and results revealed that SRL training programs can effectively enhance academic performance (*g* = 0.37), motivation (*g* = 0.35), regulation strategies (e.g., metacognitive strategies (*g* = 0.40), and resource management strategies (*g* = 0.39)) among university students. However, this meta-analysis was restricted to university students. Similarly, Jansen et al.’s (2019) meta-analysis also tested the effectiveness of SRL interventions for university students. Zheng (2016) examined the functions of scaffolding in relation to both K–12 and university students, and found a moderately positive effect on academic performance (*g* = 0.438). However, this meta-analysis only included 29 studies, and most studies were focused on (biased toward) higher education. Several other meta-analyses also didn’t simultaneously concern K12 and higher education, such as Dignath et al. (2008) and de Boer et al. (2018), both of which examined only primary and secondary school. Thus, there is a need to test the effectiveness of regulated learning scaffolding using a larger database that includes more diverse student samples.

Further, most previous meta-analyses have not examined the effectiveness of various types of regulated learning scaffolding, such as scripts, group awareness tools, intelligent pedagogical agents, and composite tools, at the macro level, instead focusing on the mechanisms, functions, delivery forms, and number of scaffolding (Zheng, 2016; Guo, 2022), that is, micro-level issues. However, different types of macro-level scaffolding have specific micro-level characteristics. Therefore, it is necessary to view regulated learning scaffolding from a macro-level perspective. Besides, we can further explore which type of scaffolding is most effective for different outcome variables. Moreover, there has been no meta-analysis of SSRL scaffolding, despite previous studies finding that SSRL is positively linked to academic performance (Panadero and Järvelä, 2015). Most meta-analyses only focused on SRL scaffolding, such as Theobald (2021) and Guo (2022). Thus, given the high degree of relevance of SSRL to academic performance, it is also important to test the effectiveness of scaffolding in relation to SSRL.

### 2.3. Moderators of regulated learning scaffolding’ effectiveness

Characteristics included in previous regulated learning review studies have been incorporated into this review as moderators, and are described below.

#### 2.3.1. Types of regulated learning scaffolding

*Scripts* are a critical component in students’ learning (see Chi et al., 1994, 2001). Several studies have found that when students learn about complex topics with scripts, they are better able to regulate their learning and gain a conceptual understanding of the topic (Hill and Hannafin, 1997; Greene and Land, 2000; Azevedo and Cromley, 2004). *Group awareness* is defined as up-to-date information obtained by an individual in the group on the activities and situations of others that can be used for coordinating and completing a part of a group task (Yilmaz and Yilmaz, 2019). Group awareness tools can be used to increase a group’s collective actions and visualize social interactions (Kreijns et al., 2002), thereby increasing the effectiveness of collaboration (Janssen and Bodemer, 2013), and providing students, particularly those with a low level of SRL, with the opportunity to observe and emulate role models (Lin et al., 2016). *Intelligent pedagogical agents* are actuated by users in a virtual environment and have been developed for educational purposes (Yilmaz and Cakmak, 2011). A review of previous studies revealed that the use of a pedagogical agent in online learning environments had a positive effect on learning processes and outcomes such as motivation (Dinçer and Doğanay, 2017), achievement (Yilmaz and Cakmak, 2012), and behavioral intentions (Guo and Goh, 2016). Several studies have confirmed the positive effects of pedagogical agents on self-regulation skills and metacognitive awareness (Molenaar et al., 2011; Yilmaz et al., 2018). However, another study found that metacognitive scaffolding provided by a pedagogical agent did not have a significant effect on either group performance or individual domain knowledge (Molenaar et al., 2011). Thus, it is necessary to examine the effects of these regulated learning scaffolding on regulation levels, regulation strategies, and academic performance in an effort to determine which regulated learning scaffolding has the greatest effect.

#### 2.3.2. Cooperation

In cooperative learning, students share responsibilities, ideas, and thoughts to promote metacognitive reflection and motivation (Chiu and Kuo, 2009). Prior meta-analyses have reported inconsistent results including a negative impact (e.g., Boer et al., 2014) and no impact for primary school students but a positive impact on comprehension and conceptual understanding for secondary school students (e.g., Dignath et al., 2008). A meta-analysis of university students by Theobald (2021) found that collaborative learning elevated SRL training effects on metacognitive strategies. However, it remains unclear whether the effects would be greater if students worked collaboratively rather than individually.

#### 2.3.3. Academic subject

Each subject area constitutes a distinct context that can potentially influence students’ SRL (Wolters and Pintrich, 1998; Patrick et al., 2007). Further, students’ use of cognitive and metacognitive strategies might be based on the learning domain (Wolters and Pintrich, 1998). However, whether the academic subject shapes regulated learning and its association with achievement has received relatively little attention (e.g., Wolters and Pintrich, 1998). Instead, most studies exploring the outcomes of regulated learning have focused on a single subject area (Dent and Koenka, 2016). Therefore, integrating these findings enables us to test whether the academic subject has a moderating effect.

#### 2.3.4. Grade level

Self-regulated learning has been described as an “inherent aspect of learning” (Winne, 1995, p. 186), and students’ ability to undertake SRL develops with age (Paris and Newman, 1990). In elementary school, students have only a vague understanding of academic tasks (e.g., Meyers and Paris, 1978), and rarely monitor or reflect on their task performance (e.g., Skinner et al., 1988). Following the transition to middle school, variations in achievement are more closely related to variations in SRL (Brookhart, 1994; McMillan and Workman, 1998). SRL continues to develop during adolescence, with metacognitive monitoring and reflection improving significantly (Keating, 1990; Ryan and Pintrich, 1997). It can be seen that students’ grades are closely related to their regulated learning ability. Therefore, a meta-analysis can be used to explore how regulation levels, regulation strategies, and academic performance are influenced by grade level and academic subject.

### 2.4. The current meta-analysis

In current meta-analysis, we aimed to investigate the impact of regulated learning scaffolding on regulation strategies and academic performance. Empirical studies in the past decade that examined various regulated learning scaffolding’s effect were evaluated. The findings offered insights into the overall effectiveness of regulated learning scaffolding as well as the respective roles of SRL scaffolding and SSRL scaffolding. In addition, they provided directions for determining the most effective scaffolding for different outcome categories and key factors to consider when implementing scaffolding in the future. Specifically, three research questions were raised in this study:

1. What is the overall effect of regulated learning scaffolding?
2. Which kinds of regulated learning scaffolding are most effective in improving regulated learning (SRL/SSRL) and learning outcomes (regulation strategies and academic performance)?
3. How are the effects moderated by cooperation, grade level, and academic subject?

The first research question examined the effect of regulated learning scaffolding on overall outcome categories, as well as on the sub-category (type of regulated learning and learning outcome). We predicted that regulated learning scaffolding would have a moderate effect on both overall outcome categories and the sub-category.

The second research question examined more specifically the effects of different type of regulated learning scaffolding (scripts, group awareness, intelligent pedagogical agents, composite tool), and clarified what is the most effective scaffolding for different outcome categories. We anticipated that overall, composite tools would have the greatest effect. For SRL and SSRL, the most useful scaffolding may be group awareness tools and composite tools, respectively. In terms of learning outcomes, scripts and intelligent pedagogical agents would have the greatest effect on regulation strategies and academic performance, respectively.

The third research question involved moderator analysis. We assumed that regulated learning scaffolding would have the greatest impact on primary school learners, collaborative learners, and natural science learners, respectively.

## 3. Method

### 3.1. Literature search

The meta-analysis followed the Preferred Reporting Items for Systematic Reviews and Meta-Analyses (PRISMA) guidelines for reporting meta-analytical findings (Moher et al., 2009). The PRISMA consists of a 27-item checklist (such as, title, abstract, method, results, discussion and so on) and a four-phase flow diagram (identification, screening, eligibility and included). Research should strictly follow this process to improve the reporting of systematic reviews and meta-analyses. The identification of relevant studies was conducted via the online databases Web of Science, Elsevier Science Direct, and Proquest, and covered articles published in English from January 2000 to December 2021. Review articles and conference papers were excluded. The following search terms were used:

Abstract: (self-regulated learning OR socially shared regulated learning OR regulat* learning OR SRL OR SSRL) AND abstract:(tool OR scaffold OR group awareness OR script OR pedagogical agent) AND abstract:(achievement OR performance OR level OR strategy).

### 3.2. Criteria for inclusion and exclusion

First, because the meta-analysis focused on the ways in which regulated learning scaffolding affects students’ academic performance and regulation strategies, at least one type of regulated learning scaffolding had to be used and the studies had to report on at least one of the following outcomes: students’ regulation strategies and academic performance. Studies that didn’t focus on regulated learning scaffolding or didn’t pay attention to these two types of outcome variables will be excluded.

Moreover, the studies had to target in-school or online students, including pre-K children, primary school students, junior and senior high school students, and higher education students. Studies focused on pre-service teachers, children with disabilities, or other adult learners were excluded.

Only empirical studies were selected, and they had to include a control group and an experimental group. Excluding surveys, one-group pretest-posttest designs, and qualitative studies. In addition, studies had to contain sufficient information (N, M, SD) to enable the computation of effect sizes.

### 3.3. Selection of studies

**Figure 1:**
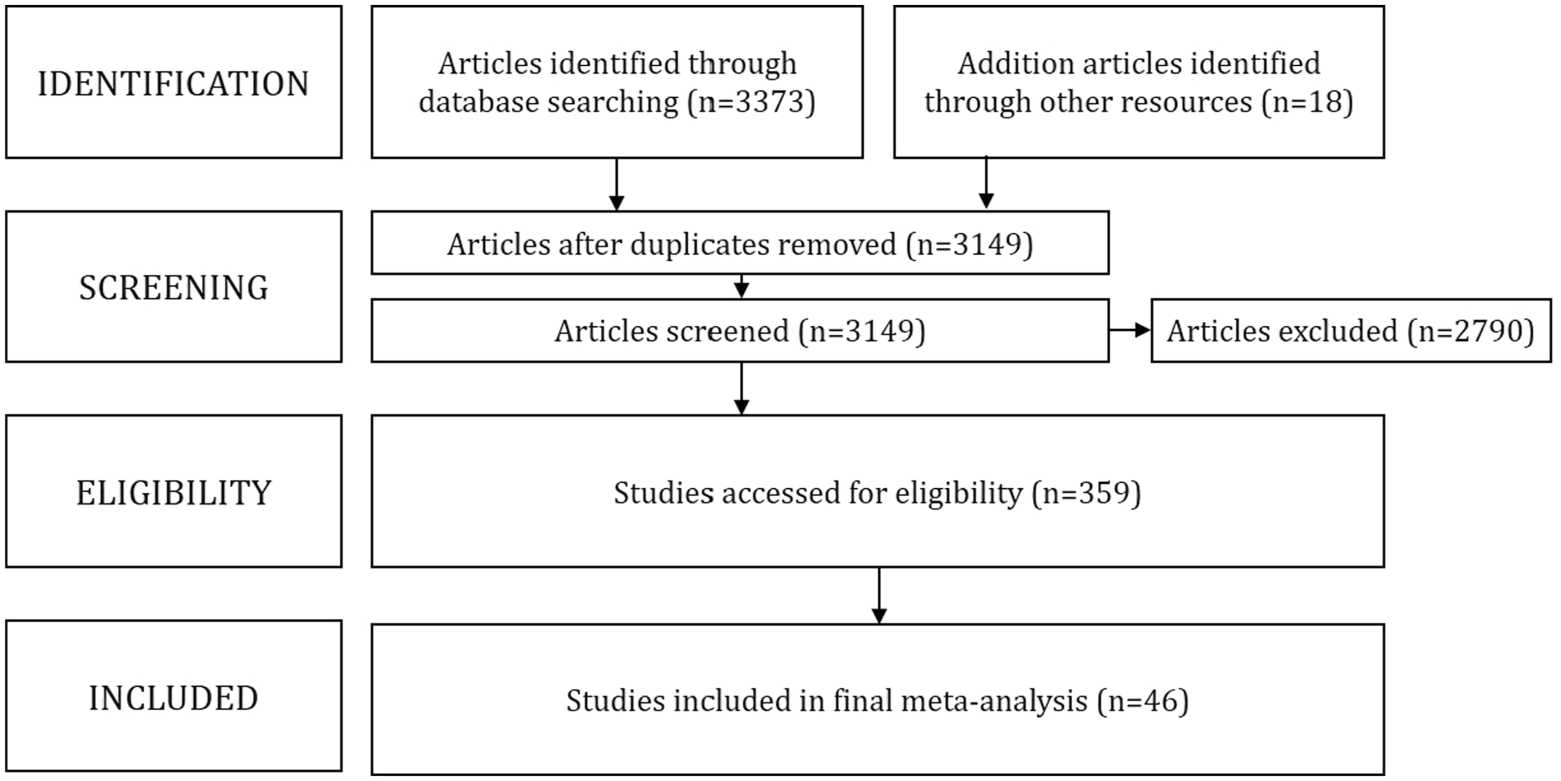
Literature selection process.

Figure 1 shows the flow diagram for the literature selection process. In the first step, 3,391 articles were identified from the database search (*n* = 3,373) or a backward and forward search of the literature (*n* = 18). Then we removed duplicate studies (*n* = 242). This preliminary search yielded 3,149 articles. After collecting articles, we reviewed titles and abstracts to remove studies that didn’t fit any of the above three inclusion criteria, resulting in 2,790 articles being deleted, and 359 articles being retained for the next step. Next, we thoroughly scrutinized the full texts of these articles. Then, 313 articles that did not meet the requirements of the meta-analysis were deleted. Thus, 46 studies met all inclusion criteria and were included in the meta-analysis.

### 3.4. Coding of outcome categories and moderating variables

Two outcome categories were set: type of regulated learning and learning outcomes. The type of regulated learning was classified into SRL and SSRL, and the learning outcomes were academic performance and regulation strategies. Based on the classification of regulation strategies in previous studies, we classified regulation strategies into three lower-level categories. *Cognitive strategies* refer to how to learn and integrate new knowledge into existing structures, such as rehearsal, elaboration, and organization (Weinstein and Mayer, 1985). *Metacognitive strategies* refer to the monitoring and controlling of the application of cognitive strategies (Flavell, 1979), and encompass goal-setting, planning, monitoring, and reflection. *Resource management strategies* refer to the integration of internal (attention, emotion, and motivation) and external (environment, time, and management) resources (Pintrich, 1999). Numerous studies have been conducted on learners’ use of regulation strategies. For example, Dignath et al. (2008) found that self-regulation strategies can be improved through training. Järvelä et al. (2008) pointed out that students adopt and activate new motivation strategies to fit specific challenges in socially shared learning, and strong shared regulation groups are more likely to activate cognitive and metacognitive strategies (Järvelä et al., 2013). Outcomes other than academic performance were mostly assessed by questionnaire.

Four moderating variables were coded. *Types of regulated learning scaffolding.* We divided the regulated learning scaffolding into four categories: (1) scripts, (2) intelligent pedagogical agents, (3) group awareness tools, and (4) composite tools. We then coded whether the regulated learning scaffolding were used consistently. *Cooperation.* Based on whether students had adopted collaborative learning, we divided learning into two categories: (1) individual learning, and (2) collaborative learning. *Academic subject.* Four subject areas were considered: (1) social sciences, (2) natural sciences, (3) engineering and computer science, and (4) language acquisition. *Grade level.* We divided education into three levels: (1) primary education, (2) secondary education, and (3) higher education.

**Figure 2:**
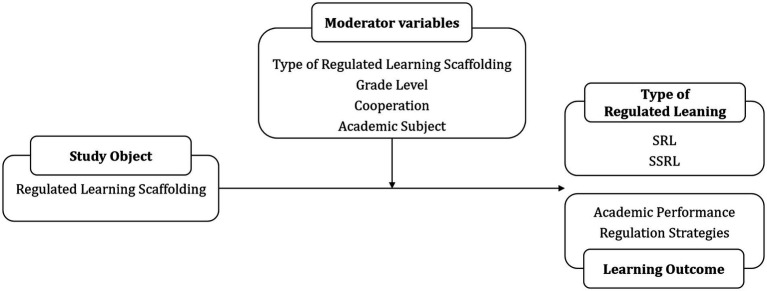
Overview of study objects, outcome categories, and moderating variables.

A detailed overview of the coding of outcome categories and moderating variables is presented in Table 1 and Figure 2.

**Table 1:** Overview of outcome categories and moderating variables included in the meta-analysis.

Category	Variable
Outcome categories	
Type of regulated leaning	SRL
	SSRL
Learning outcome	Academic performance
	Regulating strategies
	Cognitive strategies
	Metacognitive strategies
	Planning & goal setting
	Monitoring
	Reflection & evaluation
	Resource management strategies
Moderator variables	
Type of regulated learning scaffolding	Script
	Intelligent pedagogical agent
	Group awareness
	Composite tool
Grade level	Primary education
	Secondary education
	Higher education
Cooperation	Individual learning
	Collaborative learning
Academic subject	Social sciences
	Natural sciences
	Engineering and computer
	Language acquisition

Each study was independently coded by two raters based on the coding schemes and the inter-rater reliability calculated by Cohen’s kappa was 0.89, which was regarded as reliable. In response to discrepancies, the two raters discussed and resolved the problem.

### 3.5. Data analysis

To calculate the effect sizes, we used comprehensive meta-analysis (CMA) software developed by Biostat. The effect size was calculated based on the sample sizes, mean outcome scores, and standard deviations for both the experimental group and the control group (standardized mean differences). If one study adopted multiple tests to examine academic performance, the effect sizes were averaged to obtain one representative effect size for each study using CMA software. However, Cohen’s d has a slight upward bias, especially in small samples. Hedges (1981) proposed removing this bias by using correction factor J. Thus, we set Hedge’s g as the main indicator to determine the size of the effect, with effect sizes of 0.30, 0.50, and 0.80 corresponding to a small, medium, or large effect, respectively.

First, we combined the overall effect of regulation using a random-effects model because we assumed that the size of the effect could vary from study to study (Borenstein et al., 2011). The confidence interval (CI) of the pooled effect size was set at 95%. In addition, publication bias was checked using the funnel plot and Egger’s linear regression test. Then, we compared the size of the effect of scaffolding on different types of regulated learning and learning outcomes. Next, moderator analysis was conducted to examine the effects of different types of regulated learning scaffolding on the subgroups (Karadag et al., 2015), and obtained the following information: (a) the mean, standard errors, and 95% confidence intervals of the effect size for each category, and *p* values to indicate whether each effect size was significantly different from zero; and (b) the p values to indicate whether the effect size of each category was significantly different from the effect size of another category. Finally, we ran a simultaneous meta-regression with multiple independent variables to examine how different characteristics explain the effects of regulated learning scaffolding. In this analysis, the effect size was the dependent variable, and grade, academic subject, and cooperation were the independent variables.

## 4. Results

### 4.1. Descriptive results

We examined 46 peer-reviewed journal articles published between 2010 and 2021, of which 37 examined the effects of scaffolding on SRL activities and nine examined the effects of scaffolding on SSRL activities. Overall, 138 effect sizes were reported, of which 34 related to academic performance and 104 related to regulation strategies.

The heterogeneity tests for regulation strategies and academic performance were significant and there was a moderate amount of variance in the effect sizes (*Q* = 846.502, df = 137, *p* = 0.000; *I*^2^ = 83.82). Thus, the hypothesis of homogeneity was rejected, suggesting the necessity of moderator analyses in an effort to ascertain the variables that might explain the heterogeneity and to select a random effects model. Using a random effects model, these analyses showed that regulated learning scaffolding had moderately positive effects. The average weighted effect size was Hedges’ g = 0.587 (SE = 0.056). Table 2 presents the number of studies (k), effect size (g), standard error (SE), variance, confidence intervals, z value, p value, and test of heterogeneity in effect size.

**Table 2:** Overall effect sizes.

	k	g	SE	Variance	95%CI		Z	*p*	Heterogeneity		
					Lower	Upper			Q	df	*p*
Random	138	0.587	0.056	0.003	0.476	0.692	10.412	0.000	846.502	137	0.000

K, Number of effect sizes; g, Hedges’ g effect size; SE, standard error; CI, confidence interval.

Regarding the type of regulated learning, we calculated the overall effect for SRL and SSRL (Table 3). The average weighted effect size was Hedges’ g = 0.603 (SE = 0.066) for SRL and *g* = 0.530 (SE = 0.108) for SSRL.

**Table 3:** Effect sizes by type of regulated learning.

	k	g	SE	Variance	95%CI		Z	*p*	Heterogeneity test
					Lower	Upper			
SSRL	34	0.530	0.108	0.012	0.319	0.742	4.920	0.000	*Q* = 0.335
SRL	104	0.603	0.066	0.004	0.475	0.732	9.185	0.000	*p* = 0.562

K, Number of effect sizes; g, Hedges’ g effect size; SE, standard error; CI, confidence interval.

Regarding learning outcomes, the greatest effect size was associated with regulation strategies (*g* = 0.617, *p* < 0.001), followed by academic performance (*g* = 0.500, *p* < 0.001). In terms of specific regulation strategies, the largest average effect sizes were obtained for resource management strategies (*g* = 0.826, *p* < 0.001) and metacognitive strategies (*g* = 0.546, *p* < 0.001), while the smallest effect size was in relation to cognitive strategies (*g* = 0.326, *p* < 0.1). Regarding specific metacognition regulation strategies, the effect sizes ranged from 0.369 (monitoring) to 0.769 (reflection and evaluation) (Table 4).

**Table 4:** Effect sizes by learning outcome.

	k	g	SE	Variance	95%CI		Z	*p*	Heterogeneity
					Lower	Upper			
Academic performance	34	0.500	0.102	0.010	0.299	0.700	4.882	0.000	Q = 0.923
Regulation strategies	104	0.617	0.068	0.005	0.485	0.750	9.144	0.000	*p* = 0.337
Cognition strategy	21	0.326	0.063	0.004	0.202	0.450	1.898	0.058	Q = 3.501
Recourse management strategy	13	0.826	0.077	0.006	0.674	0.977	3.879	0.000	
									*p* = 0.321
Metacognition strategy	51	0.546	0.039	0.002	0.470	0.622	6.547	0.000	
Planning and goal setting	23	0.649	0.163	0.026	0.331	0.968	3.992	0.000	Q = 6.903
Monitoring	16	0.369	0.075	0.006	0.221	0.516	4.893	0.000	
									*p* = 0.067
Reflection and evaluation	11	0.769	0.219	0.048	0.340	1.198	3.514	0.000	

K, Number of effect sizes; g, Hedges’ g effect size; SE, standard error; CI, confidence interval.

### 4.2. Publication bias

**Figure 3:**
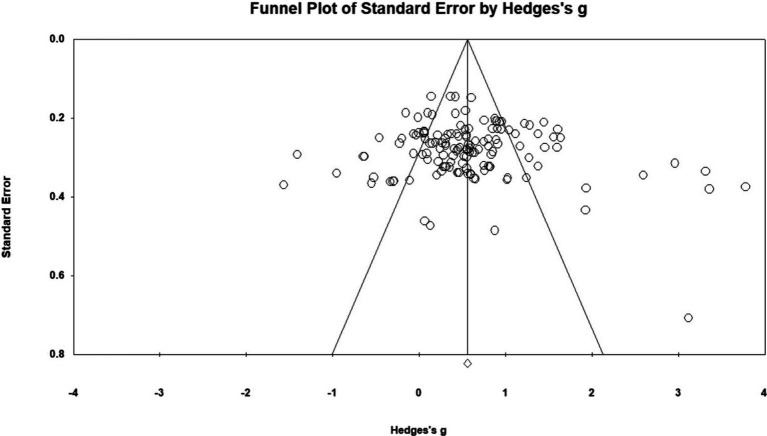
Funnel plot of standard errors by Hedges’ g effect sizes.

Studies with larger samples and significant results are more likely to be published than those with smaller samples and non-significant results (Borenstein et al., 2011). This results in publication bias, which may lead to bias in the sample set selected for inclusion in a meta-analysis. In this study, examination of the funnel plot (see Figure 3) showed that the effect sizes were distributed symmetrically around the mean effect size, with no concentration of effect sizes on either side. The results of Egger’s regression test for funnel plot asymmetry rejected the presence of publication bias (*t* = 1.27, *p* = 0.10). Taken together, the funnel plot and Egger’s regression test results suggested that the observed overall effect sizes were not an artifact of publication bias.

### 4.3. Regulated learning scaffolding’ effects by outcomes

Regarding the effects of different types of regulated learning scaffolding, the greatest effect size was associated with composite tools (*g* = 0.700, *p* < 0.001), followed by group awareness tools (*g* = 0.604, *p* < 0.001), intelligent pedagogical agents (*g* = 0.542, *p* < 0.01), and scripts (*g* = 0.525, *p* < 0.001) (Table 5).

**Table 5:** Overall effectiveness of various regulated learning scaffolding.

	k	g	SE	Variance	95%CI		Z	*p*	Heterogeneity test
					Lower	Upper			
Script	69	0.525	0.079	0.006	0.370	0.680	6.628	0.000	Q = 1.977
Group awareness	26	0.604	0.165	0.027	0.280	0.927	3.653	0.000	
Intelligent pedagogical agent	10	0.542	0.203	0.041	0.144	0.939	2.670	0.008	*p* = 0.578
Composite tool	33	*0.700*	0.099	0.010	0.506	0.895	7.062	0.000	

Italics indicate that it is the largest value. K, Number of effect sizes; g, Hedges’ g effect size; SE, standard error; CI, confidence interval.

#### 4.3.1. Scaffolding’ effects by type of regulated learning

As shown in Table 6, the impact of various scaffolding varied between SRL and SSRL. For SRL, group awareness tools had the greatest effect (*g* = 1.298, *p* < 0.001), while for SSRL, composite tools had the greatest effect (*g* = 0.871, *p* < 0.001).

**Table 6:** Scaffolding’ effects by type of regulated learning.

	SRL				SSRL			
	k	g	SE	*p*	k	g	SE	*p*
Script	67	0.528	0.082	0.000	2	0.439	0.175	0.012
Group awareness	8	*1.298*	0.381	0.001	18	0.303	0.136	0.026
Intelligent pedagogical agent	10	0.542	0.203	0.008				
Composite tool	19	0.602	0.113	0.000	14	*0.871*	0.192	0.000

Italics indicate that it is the largest value. K, Number of effect sizes; g, Hedges’ g effect size; SE, standard error.

#### 4.3.2. Scaffolding’ effects by learning outcome

The effects of regulated learning scaffolding also varied in relation to different learning outcomes (see Tables 7–9). For academic performance, intelligent pedagogical agents were the most useful tool, with an effect size of 0.558 (*p* < 0.01), while for regulation strategies, most of the regulated learning scaffolding had a moderate effect (*g* > 0.50), with composite tools having the greatest effect (*g* = 0.750, *p* < 0.001). In terms of sub-categories, all regulated learning scaffolding had a moderate effect in relation to cognition strategies (*g* < 0.40), with scripts having the greatest effect (*g* = 0.406, *p* < 0.1). Surprisingly, the effect of intelligent pedagogical agents was negative (*g* < 0.00). In terms of resource management strategies and metacognitive strategies, group awareness tools (*g* = 1.844, *p* < 0.01) and composite tools (*g* = 0.737, *p* < 0.01) had the greatest effects. Regarding specific metacognitive strategies, composite tools, intelligent pedagogical agents and group awareness tools, respectively, had the greatest effect on planning and goal-setting, monitoring, and reflection and evaluation.

**Table 7:** Scaffolding’ effects by learning outcome.

	Academic performance				Regulation strategies			
	k	g	SE	*p*	k	g	SE	*p*
Script	18	0.518	0.146	0.000	51	0.529	0.095	0.000
Group awareness	5	0.267	0.350	0.445	21	0.682	0.190	0.000
Intelligent pedagogical agent	4	*0.558*	0.504	0.268	6	0.544	0.183	0.003
Composite tool	7	0.501	0.115	0.000	26	*0.750*	0.125	0.000

Italics indicate that it is the largest value. K, Number of effect sizes; g, Hedges’ g effect size; SE, standard error.

**Table 8:** Scaffolding’ effects by sub-category.

	Cognition strategy				Recourse management strategy				Metacognition strategy			
	k	g	SE	*p*	k	g	SE	*p*	k	g	SE	*p*
Script	15	*0.406*	0.241	0.092	6	0.525	0.212	0.014	23	0.494	0.113	0.000
Group awareness	5	0.232	0.216	0.283	3	*1.844*	0.648	0.004	9	0.572	0.312	0.067
Intelligent pedagogical agent	1	−0.001	0.238	0.997					2	0.406	0.170	0.017
Composite tool					4	0.475	0.138	0.001	17	*0.737*	0.163	0.000

Italics indicate that it is the largest value. K, Number of effect sizes; g, Hedges’ g effect size; SE, standard error.

**Table 9:** Scaffolding’ effects by metacognitive strategy.

	Planning and goal setting				Monitoring				Reflection and evaluation			
	k	g	SE	*p*	k	g	SE	*p*	k	g	SE	*p*
Script	10	0.486	0.246	0.048	8	0.437	0.127	0.001	4	0.581	0.176	0.001
Group awareness	6	0.183	0.182	0.313	1	0.216	0.244	0.376	2	*1.920*	1.035	0.064
Intelligent pedagogical agent					1	*0.439*	0.241	0.069	1	0.372	0.240	0.121
Composite tool	7	*1.314*	0.308	0.000	6	0.282	0.114	0.014	4	0.519	0.279	0.063

Italics indicate that it is the largest value. K, Number of effect sizes; g, Hedges’ g effect size; SE, standard error.

### 4.4. Moderator analyses

To facilitate a meaningful interpretation of the results, we included the other moderating variables in the meta-regression. As can be seen from Table 10, the categorical variables were dummy coded and used as predictors in the meta-regression. For example, of the three grade levels (primary school, junior and senior high school, and higher education), we used higher education as the reference variable and included the other two predictors in the meta-regression.

**Table 10:** Results of meta-regression of moderating variables.

Model		Full model			
Variables coded	Predictors	Regression coefficient	Standard error	*p*	
	Intercept	−0.199	0.175	0.258	
Grade level (reference: higher education)	Primary school	0.723	0.204	0.000	*Q* = 13.46, *p* = 0.001
	junior and senior high school	−0.134	0.168	0.427	
Academic subject (reference: engineering and computer)	Social science	0.492	0.171	0.004	*Q* = 15.78, *p* = 0.001
	Natural science	0.747	0.195	0.000	
	Language acquisition	0.582	0.188	0.002	
Cooperation (reference: individual learning)	Collaborative learning	0.531	0.122	0.000	

In the full model, only junior and senior high school was not statistically significant (*p* = 0.427 > 0.1). The effect size of primary school was larger by a 0.723 standard deviation unit than the effect size of higher education. The effect sizes of social science, natural science, and language acquisition were greater than those of engineering and computer science. When collaborative learning was included, the effect size was larger than that when only individual learning occurred by 0.531 standard deviation unit.

## 5. Discussion

### 5.1. Overall effect

Consistent with our hypothesis, the findings of this meta-analysis of 46 studies indicate that regulated learning scaffolding has a moderately positive effect (*g* = 0.587) on regulated learning. This is consistent with the results of a previous study by Schmid et al. (2014), who found that the overall weighted average effects of technology use on achievement were significant, and is also in line with the results of a previous meta-analysis that found a positive overall effect of SRL scaffolding on academic performance (Zheng, 2016). One difference is that the current study focused on regulated learning scaffolding rather than training programs or scaffolding mechanisms, enabling us to examine the effects from a macro perspective.

We further explored the effects of scaffolding on SRL and SSRL, and as anticipated, provided the first evidence of the effects of scaffolding on SRL (*g* = 0.603), supporting the findings of previous meta-analyses. As Theobald (2021) pointed out, SRL training programs could be used to enhance students’ academic performance, SRL strategies, and motivation. This corroborated the findings of a study by Stevenson et al. (2017), which confirmed the positive effect of concept mapping technologies on SRL. However, one distinction is that the current study focused on four types of regulated learning scaffolding (i.e., group awareness tools, scripts, intelligent pedagogical agents, and composite tools), which enabled us to obtain a more comprehensive understanding of the effects of various technologies. Another distinction is that this meta-analysis adds to previous research in this area by showing that regulated learning scaffolding also has a positive effect on SSRL (*g* = 0.530). Numerous studies have demonstrated that learners often fail to achieve socially shared regulation during collaborative learning (Zimmerman and Schunk, 2011; Kirschner and Erkens, 2013) because it is more difficult to regulate at the group level than at the individual level (Winne et al., 2013). Thus, external support is essential for facilitating SSRL (Järvelä et al., 2015). The results of this study support the use of regulated learning scaffolding for SSRL. Zheng et al. (2017) proposed and developed a collaborative learning tool using a socially shared regulation approach, and measured its effectiveness in relation to participants’ learning achievement, group performance, and socially shared regulation frequency.

We also examined the effects of regulated learning scaffolding on learning outcomes (academic performance and regulation strategies). Consistent with predictions, our results supported the findings of previous meta-analyses that regulated learning scaffolding had a moderate effect on academic performance (Zheng, 2016) and a small to moderate effect on regulation strategies (Theobald, 2021). The results showed that regulated learning scaffolding had the greatest effect on regulation strategies. This confirmed the finding of Jansen et al. (2019), who found that the effects of interventions were greater in relation to regulation strategies than in relation to academic performance. One possible reason is that regulation strategies moderate the relationship between regulated learning scaffolding and academic performance (Jansen et al., 2019), and thus regulated learning scaffolding possibly weaken some effects on academic performance. Another possible reason is that regulation strategies are difficult to measure. Some researchers have argued that self-reporting and questionnaires, which have been used to measure regulation strategies in numerous studies, are not always effective (Azevedo and Cromley, 2004). Thus, this inaccurate measure probably resulted to the high value of the regulation strategies. Regarding the sub-categories, the effects of regulated learning scaffolding on cognitive strategies were smaller than those on resource management and metacognitive strategies. This result is consistent with that of Theobald (2021), and might be because students’ cognition is difficult to change over a short study period, which means more extensive intervention and time should be taken. In terms of metacognitive strategies, the results showed that regulated learning scaffolding had a positive effect on planning and goal-setting, as well as reflection and evaluation, but had little effect on monitoring. Therefore, future research should investigate ways to enhance monitoring.

### 5.2. The effect of regulated learning scaffolding

In terms of different types of regulated learning scaffolding effects, consistent with hypothesis, we found that composite tools had the greatest overall effect. Composite tools integrate numerous features of other tools, such as question guides, visual diagrams, constructive modules, templates, animated or human-like communication, and visual and auditory hints (Manlove et al., 2006; Zheng et al., 2017). Various functions can be arranged to appear at the right moment to support students’ diverse needs, encourage group participation, remind students to regulate and monitor themselves, stimulate their interest in learning with animations or diagrams, and promote their cognitive and metacognitive development with a variety of materials (Bellhäuser et al., 2016). Further, the cognitive theory of multimedia learning states that student learning is enhanced by the presentation of both words and pictures rather than only words (Meyer, 2009). Therefore, when using composite tools, students can obtain access to more materials and integrate visual and verbal representation, thereby simultaneously promoting students’ regulated learning through both channels. This might explain why composite tools had the greatest effect. Below, results are discussed separately for two aspects: the type of regulated learning and the learning outcome.

#### 5.2.1. The effect of scaffolding by type of regulated learning

Regarding the type of regulated learning, as expected, the most useful scaffolding for SRL is the group awareness tool. Pintrich (2000) stated that learners could only undertake SRL effectively if they were motivated and persistent in their learning activities. Group awareness provides maximum sensory stimulation to students without cognitive load. Expressed in the form of pictures, diagrams, and text, it allows students to observe their own progress and status, as well as those of their peers, their own group, and other groups at any time, and reminds them to cooperate with each other. Pintrich (1999) also noted that social comparison/peer comparison led students to employ more cognitive and self-regulatory strategies, and that group awareness provided more opportunities and conditions for social comparison than other scaffolding. In addition, Zimmerman’s (2000) model included four aspects of developmental self-regulation: observation, emulation, self-control, and self-regulation. Group awareness fits this pattern precisely. Firstly, students obtain a basic understanding of SRL by observing the learning behavior trajectory of others (observation). Then, students can emulate the role models they have observed, comparing themselves with others and gradually practicing the skills independently (emulation, self-control). Finally, students gradually acquire self-regulation skills through repeated practice (self-regulation) (Lin et al., 2016).

While the composite tool had the greatest effect on SSRL, Järvelä et al. (2015) emphasized three design principles for supporting SSRL: awareness, social space, and sharing in interaction, prompting regulation. These principles suggest that scaffolding supporting SSRL should both enhance learners’ awareness of their own and others’ learning processes, that is, they can jointly set goals and monitor their learning progress, and provide a shared space in which they can set group norms, interaction rules, and roles to promote collaboration and socioemotional interaction, as well as enabling them to identify challenging learning situations and typical learning patterns, in other words, to focus on the process of collaboration in real time. Based on such principles and features, there is no doubt that the composite tool is the most compatible and effective option.

#### 5.2.2. The effect of scaffolding by learning outcome

Regarding learning outcomes, a *script* is the most effective scaffolding for cognitive strategies. Scripts can stipulate the sequence and type of learning activities, and collaboration roles to help group members collaborate and solve problems (Wang et al., 2017). Scripts can take many forms, such as a teacher’s oral presentation, role assignment, question prompts, peer feedback, and worked examples. They are structured, elaborative, and enlightening, and can take the form of social scripts, content-oriented (epistemic) scripts, communication-oriented (collaborative) scripts, and metacognition scripts (Noroozi et al., 2012). Changes in cognitive strategies are difficult to achieve, and must be accompanied by deep processing of new knowledge and connections to prior knowledge, requiring the long-term support of procedural tools. Given the advantages of scripts, this is probably why they are the most effective scaffolding in relation to cognitive strategies. Previous studies have found that scripts enable students to anchor newly acquired knowledge within prior knowledge (Broadbent et al., 2020), stimulate the activation of learning strategies, and enhance deeper processing of information (Bannert et al., 2015).

*Group awareness* is the most useful scaffolding for resource management strategies, as well as reflection and evaluation. Group awareness tools enable the interactive activities of each team to be visualized, including the number of individual contributions, evaluations, replies, and “likes” (Lin, 2018). Visualization strengthens social interaction and communication by enhancing motivation, triggering reflection, and monitoring individual contributions (Janssen et al., 2007), enabling the formation of a learning community (Hu et al., 2002). This mechanism enables group awareness to help students to realign their tactics for subsequent learning activities, identify problems, reflect on the entire learning process, manage their time, and foster motivation (Janssen et al., 2007; Järvelä et al., 2013; Lin, 2018). Thus, resource management (e.g., effort management, motivation regulation, and time and study management), as well as reflection and evaluation, are best facilitated by group awareness tools.

*Intelligent pedagogical agents* are the most effective scaffolding for academic performance and monitoring. One reason is that agents are designed to scaffold learning processes in a timely manner by providing feedback and prompts in response to learners’ behavior, progress, and self-evaluation (Duffy and Azevedo, 2015). Therefore, agents can monitor the overall learning process by prompting students to set goals, paying attention to time, progress, and group participation, and facilitating self-reflection, resulting in improved academic performance and monitoring. In addition, Jones et al. (2018) found that higher levels of personalization and adaptive scaffolding had a greater impact on regulation strategies and learning gains. There is no doubt that intelligent pedagogical agents are more adaptive than the other scaffolding. However, they have a negative effect on cognitive strategies. One reason is that it is difficult for agents to enhance cognitive strategies (Theobald, 2021). Another reason might be the small number of articles which is not representative.

*Composite tools* have the greatest effect on regulation strategies, metacognition, and planning and goal-setting. We predicted that the most useful scaffolding for regulation strategies may be scripts. However, results were contrary to predictions. Reasons for this may be that composite tools have multiple functions, including providing prompts to engage in metacognitive monitoring, presenting group information through graphs and tables, searching for information, making notes, and summarizing or providing video and discourse prompts (Janssen et al., 2007; Zheng et al., 2017). Supported by multiple functions, students can be motivated to set goals, perceive group member status, monitor their own progress and that of their peers, and reflect on their own performance, thereby promoting regulation strategies, metacognition, planning, and goal-setting. For example, Manlove et al. (2006) designed a tool incorporating goal lists, visualization trees, hints, prompts, cues, and templates to support the three phases of learning: planning, monitoring, and evaluation. This was consistent with Zheng et al.’s (2017) results, which demonstrated that the socially shared regulation-embedded CSCL tool (composite tool) contributed to the awareness and frequency of collective regulation. The reason for the prominence of planning and goal-setting is probably because it is the most frequently used feature of the composite tool (Manlove et al., 2006; Alvarez et al., 2022).

### 5.3. Other moderators of the effects of regulated learning scaffolding

The results of the meta-regression showed that all three moderator categories had a significant effect on learners’ regulation strategies and academic performance. Consistent with predictions, regulated learning scaffolding has a greater impact on primary school learners, collaborative learners, and social science, natural science, and language learners than on higher education learners, individual learners, and engineering and computer science learners, respectively.

#### 5.3.1. Grade level

These results are inconsistent with our general perception of the situation. One reason is that higher education students are more difficult to improve their academic performance (Hattie et al., 1996), and their regulation level, learning habits, and preferences are relatively stable, which makes it more difficult to improve their learning behavior. Conversely, younger students obtain more benefits from regulated learning scaffolding and are subject to a greater effect in relation to the use of strategies (Dignath et al., 2008), confirming the results of previous studies (Hohn and Frey, 2002). This could be because younger students are more open to acquiring new strategies (Dignath et al., 2008), and the impact of the application of regulated learning scaffolding might be more obvious, while older learners are more proficient in the use of technology-based tools, and thus are more difficult to influence. Another reason could be that this meta-analysis only included a small number of studies on primary education students, and the focus was mainly on SRL, while the number of studies on higher-education students was larger and included both SRL and SSRL.

#### 5.3.2. Cooperation

This might be because communication and interactions among learners make the use of regulated learning scaffolding more effective, thereby increasing their impact on collaborative learning, consistent with the findings of numerous previous studies. Another reason might be the limited amount of research on individual learning, which means more and more studies focus on collaborative learning.

#### 5.3.3. Academic subject

Unexpectedly, regulated learning scaffolding had less impact on engineering and computer science than on other academic fields. One reason could be that learning in engineering and computer science is largely dependent on independent inquiry and practice, while learning in other fields requires more interaction and expression, thereby increasing the effect on regulated learning. There is also the possibility that engineering and computer science students are required to be proficient in the use of regulated learning scaffolding, enabling them to largely eliminate the influence of these scaffolding and focus on the learning content, thereby reducing the influence of regulated learning scaffolding on their learning outcomes.

### 5.4. Limitations

Despite this study yielded fruitful results, it also had some limitations. First, not every article addressed all moderator variables that may have an effect on our results, so we only chose some variables that appeared in the majority of articles, such as scaffolding type, collaboration, grade level, and academic subject. That is to say, some moderator variables were not considered, such as the delivery forms of scaffolding and feedback. With the increase of empirical studies, future meta-analyses could incorporate more moderator variables. Second, the sample size of our study is somewhat limited, containing only 46 articles. Although 138 effect sizes were reported, the number divided to each subcategory is small. For example, the effect size on SSRL is only 34 and on intelligent pedagogical agents is only 10. So, due to the small number, insignificant study-level results need to be interpreted with caution. Future studies could include more articles for meta-analysis with the increase of research.

### 5.5. Implications and future research

In summary, we investigated the overall effect of regulated learning scaffolding, identified which kinds of regulated learning scaffolding (including group awareness tools, scripts, intelligent pedagogical agents, and composite tools) were most effective in improving regulated learning (SRL and SSRL) and learning outcomes (academic performance and regulation strategies), and analyzed which moderators had the greatest effects. In this section, we discuss several practical implications of our findings and provide suggestions for future research.

Given that regulated learning scaffolding has a positive impact on both regulated learning and learning outcomes, it follows that they can assist students in enhancing both their academic achievement and regulation strategies. Therefore, we suggest that practitioners should implement regulated learning scaffolding to support learners’ engagement in regulation activities, as well as their achievement. In addition, research has shown that various regulated learning scaffolding are most effective in relation to different outcome variables. For example, the most effective scaffolding for academic achievement is a script, while the most effective scaffolding for regulation strategies is a composite tool. Therefore, to support student learning, teachers should select different scaffolding based on the target outcome to maximize the impact and achieve the dual goals of promoting both academic achievement and regulation strategies.

Moreover, grade level, academic subject, and cooperation should be considered when selecting regulated learning scaffolding to improve regulation strategies and academic performance. The critical period for developing students’ regulatory skills is during primary school, when their academic performance and regulation strategies are most likely to be developed. Natural science students’ abilities can also be maximized with the support of regulated learning scaffolding, and cooperative learning can enhance their effectiveness. Thus, in the future practice of regulated learning, we should pay attention to the construction of a collaborative environment and collaborative learning activities, and use different regulated learning scaffolding based on the academic field and level of education.

Finally, because some information was missing from the studies included in this meta-analysis, a number of moderators, such as the delivery forms of scaffolding and the number of scaffolding, could not be examined, but might have contributed to variations in the effect sizes. Therefore, future studies should investigate the impact of these moderators. Further, the limited number of empirical SSRL studies meant that our meta-analysis included relatively few studies on SSRL. There is a strong link between SRL and SSRL, both of which can significantly improve the in-group climate, as well as individual academic performance, and thus future research should focus more on SSRL, exploring how regulated learning scaffolding might be used to better support SSRL, and providing more data for future meta-analyses.

## 6. Conclusion

The use of regulated learning scaffolding enhanced both SRL and SSRL, as well as academic performance and regulation strategies. This study also explored the effect of four types of regulated learning scaffolding and identified which types were most effective in improving regulated learning and learning outcomes. Results showed that overall, composite tools had the greatest effect, while the most useful scaffolding for SRL and SSRL were group awareness tools and composite tools, respectively. In terms of learning outcomes, composite tools had the greatest effect on regulation strategies, while intelligent pedagogical agents had the greatest effect on academic performance. In addition, the moderating effects of grade, academic subject, and level of cooperation were analyzed, and the results of our meta-regression showed that all three had a significant moderating effect on the impact of regulated learning scaffolding. Thus, it is suggested that the use of regulated learning scaffolding in a collaborative learning environment should be considered to promote academic performance and regulation strategies. Further experimental work is needed to clarify the contextual factors that may moderate the effectiveness of regulated learning scaffolding, but the present findings are encouraging for those looking to utilize regulated learning scaffolding to enhance learning. Given the importance of SRL and SSRL, the demand for regulated learning scaffolding will not diminish, and thus future research should pay more attention to the effects of various regulated learning scaffolding on SSRL.

## Author contributions

JS, YC, XW, and XL contributed to conception and design of the study. JS presented the initial ideas, and then our group discussed together to clarify the ideas and determine the outline. Then JS coordinated the planning, organized and analyzed the data, and wrote most of the manuscript. YC and XW collected and analyzed data and wrote part of the article separately. XL gives certain modifications. YL responsible for idea alignment and financial support. All authors contributed to the article and approved the submitted version.

## Funding

This study was supported by the National Natural Science Foundation of China (grant no: 61877003), Natural Science Foundation of Beijing Province (9222019) and International Joint Research of Faculty of Education of Beijing Normal University (ICER201903).

## Conflict of interest

The authors declare that the research was conducted in the absence of any commercial or financial relationships that could be construed as a potential conflict of interest.

## Publisher’s note

All claims expressed in this article are solely those of the authors and do not necessarily represent those of their affiliated organizations, or those of the publisher, the editors and the reviewers. Any product that may be evaluated in this article, or claim that may be made by its manufacturer, is not guaranteed or endorsed by the publisher.
